# Image-Based Auto-Focus Microscope System with Visual Servo Control for Micro-Stereolithography

**DOI:** 10.3390/mi15101250

**Published:** 2024-10-11

**Authors:** Yijie Liu, Xuexuan Li, Pengfei Jiang, Ziyue Wang, Jichang Guo, Chao Luo, Yaozhong Wei, Zhiliang Chen, Chang Liu, Wang Ren, Wei Zhang, Juntian Qu, Zhen Zhang

**Affiliations:** 1 Coal Mining Research Institute, China Coal Technology and Engineering Group Co., Ltd., Beijing 100013, China; yj-liu19@tsinghua.org.cn (Y.L.);; 2 CCTEG Intelligent Strata Control Technology (Tianjin) Co., Ltd., Tianjin 300392, China; 3 State Key Laboratory of Tribology in Advanced Equipment, Department of Mechanical Engineering, Tsinghua University, Beijing 100084, China; 4 Beijing Key Laboratory of Precision/Ultra-Precision Manufacturing Equipments and Control, Tsinghua University, Beijing 100084, China; 5 State Key Laboratory of Intelligent Mining and Strata Control, Beijing 100013, China; 6 Shenzhen International Graduate School, Tsinghua University, Shenzhen 518055, China; 7 Shenzhen Key Laboratory of Advanced Technology for Marine Ecology, Tsinghua University, Shenzhen 518055, China

**Keywords:** auto-focus, laser, visual servo control, micro-stereolithography, deep learning, machine vision

## Abstract

Micro-stereolithography (μSL) is an advanced additive manufacturing technique that enables the fabrication of highly precise microstructures with fine feature resolution. One of the primary challenges in μSL is achieving and maintaining precise focus throughout the fabrication process. For the successful application of μSL, it is essential to maintain the sample surface within a focal depth of several microns. Despite the growing interest in auto-focus devices, limited attention has been directed towards auto-focus systems in image-based auto-focus microscope systems for precision μSL. To address this challenge, we propose an image-based auto-focus microscope system incorporating visual servo control. In the optical design, a transflective beam splitter is employed, allowing the laser beam to pass through for fabrication while reflecting the focused beam on the sample surface to the microscope and camera. Utilizing captured spot images and the Foucault knife-edge test, a deep learning-based laser spot image processing algorithm is developed to determine the focus position based on spot size and the number of spot pixels on both sides. Experimental results demonstrate that the proposed auto-focus system effectively determines the relative position of the focal point using the laser spot image and achieves auto-focusing through visual servo control.

## 1. Introduction

Micro-stereolithography (μSL) is a cutting-edge fabrication technique that enables the creation of highly precise three-dimensional (3D) microstructures with resolutions in the micron or even sub-micron range [1,2,3]. In contrast to traditional stereolithography, which generally operates at the macro scale, micro-stereolithography specializes in crafting intricate designs at the micron or sub-micron level [4,5,6]. This is accomplished by employing a focused laser beam to selectively cure photosensitive resin, layer by layer, according to a predefined digital model. μSL is extensively utilized in fields such as microelectronics [7], biomedical engineering [8,9], energy storage devices [10], sensors [11], and microfluidic devices [12], where the demand for high-precision microcomponents is critical. The technique’s capability to produce complex three-dimensional microstructures with high accuracy makes it a powerful tool for both research and industrial applications.

μSL enhances traditional stereolithography by miniaturizing the optical and mechanical components to achieve higher resolutions. The process typically involves the use of an ultraviolet (UV) laser or a focused light source to cure a photopolymer resin in a highly controlled manner [13]. Each layer of the microstructure is built by selectively exposing the resin to the light source, with the stage or the light beam moving in precise increments to construct the desired 3D structure. A crucial aspect of μSL is maintaining focus at the micro-scale, as any deviation can result in defects, loss of resolution, or failure to produce the desired geometry. While the spot sizes between different layers can be maintained by a precision motion system, the spot sizes of the first layer on the reference plane determine the spot sizes of all subsequent layers. The challenge in μSL systems lies in the fact that the diameter of the focused laser spot is merely a few micrometers, with the focal depth range being only a few tens of micrometers.

Therefore, it is essential to implement laser auto-focus on the reference plane for μSL. Recently, there has been an increasing demand for automated visual servo control and inspection including in laser fabrication systems [14,15], cell microinjection [16,17], defect inspection [18], microrobotic manipulation [19,20], motion control [21], and micro-objects detection [22], etc. To equip those applications with auto-focus functions, various equipment and algorithms have been proposed based on the distances between objects and cameras, or on images captured by cameras. Depending on the measurement methods, auto-focus systems can be categorized into active [23,24] and passive [25,26] systems, each with its own advantages and limitations. The active method requires less computational power but is limited in microscopic scenarios by sensor resolution, whereas the passive method can display focused results in real time but necessitates significant data processing.

In scenarios demanding high precision, the image-based auto-focus method is preferred. Previous studies have proposed various structural and algorithmic designs for different applications. Shechtman et al. proposed a framework for pupil-plane modulation for 3D imaging applications requiring precise localization, including single-particle tracking and superresolution microscopy [27]. Haferkamp et al. developed a laser cutting detection system, where the temperature field was monitored online during the cutting process with a thermocamera [28]. Chen et al. introduced a passive auto-focus camera control system incorporating an adjustable lens with a CMOS sensor, a servo motor, an 8051 image capture microcontroller, a field-programmable gate array sharpness measurement circuit, and a pulse-width modulation controller [23]. Vo et al. presented a non-contact method for thickness measurement of a transparent plate using a laser auto-focus scanning probe [29]. Hsu et al. developed a fast auto-focus microscope system based on the astigmatic method [30]. Jeon et al. proposed a fully digital auto-focusing system with automatic focusing region selection and a priori estimated dataset of circularly symmetric point-spread functions [31]. Chen et al. introduced a real-time auto-focus algorithm combining the discrete difference equation prediction model and the bisection search method for digital still cameras [26].

Auto-focus systems for μSL have garnered considerable attention in ongoing research. While graphic-based methods offer some guidance for autofocus [32,33], they are susceptible to various disturbances, including lighting conditions, image resolution and quality, background clutter, pose and orientation, data noise, and object deformation, thereby compromising their robustness. To address this, we propose a cost-efficient image-based auto-focus system comprising only a beam splitter, a camera, a microscope, and a motor. By employing the Foucault knife-edge method, the relative focal position is ascertained through the shape and size of the laser spot detected by the camera. Furthermore, we propose a deep learning-based laser spot image processing algorithm that facilitates visual servo control, ensuring a robust autofocus process.

The main contributions of this paper are as follows:

- Auto-Focus System for μSL: We propose a real-time feedback visual servo control system that automatically focuses the laser on the reference plane during μSL, utilizing camera-captured images for precise beam alignment.
- Deep Learning-Based Image Processing Algorithm: We propose a deep learning-based algorithm that enhances laser spot focus by enclosing it within a minimum enclosing circle and employing the Foucault knife-edge test. The algorithm outperforms traditional methods by autonomously classifying focus positions based on the spot’s radius and pixel distribution.

The remainder of this paper is organized as follows: Section 2 presents the design of the auto-focus microscope system. In Section 3, the algorithms related to the collected images are examined. Section 4 discusses the construction of the prototype auto-focus microscope system and verifies its performance through experiments, followed by conclusions in Section 5.

## 2. Design of the Image-Based Auto-Focus System for the μSL System

The auto-focus system proposed in this study is aimed at the μSL system based on the compliant nano-manipulator. In this section, the μSL system, optical system, mechanical system, and control system will be described, respectively.

### 2.1. μSL System

**Figure 1:**
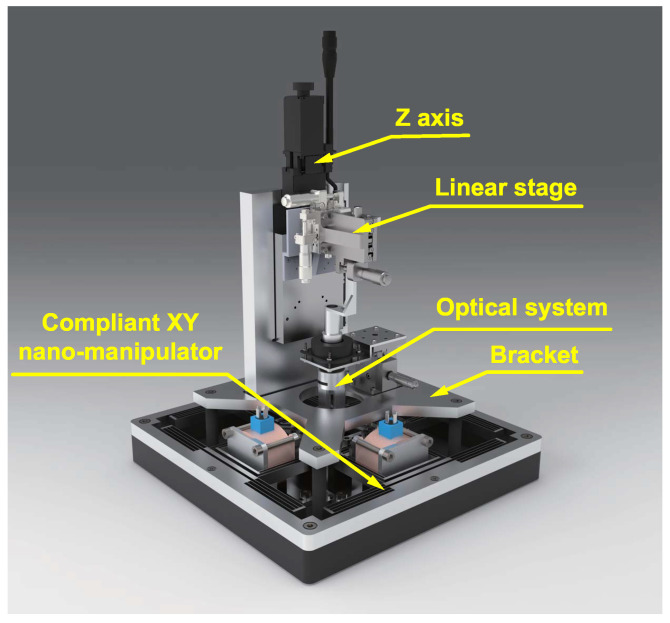
Prototype of a μSL system.

In this work, we propose an image-based auto-focus system supporting μSL. As shown in Figure 1, a μSL system is mainly composed of a self-developed compliant XY nano-manipulator [34,35,36], an optical system, and a stage. The optical system is placed on the motion stage of the compliant XY nano-manipulator through a bracket, and the upper Z axis drives the sample up and down. The compliant XY nano-manipulator is located at the bottom of the system as an enabling component for large-scale, high-precision planar motion. Its motion range can reach 2×2 mm^2^, with nano-level motion accuracy. At the same time, the Z axis drives the sample to move to ensure that the surface of the sample is in the focal depth of the laser.

The auto-focus system for the μSL system can be divided into the optical system, the mechanical system and the control system. In this section, the design of these parts will be introduced, respectively.

### 2.2. Optical System

In the μSL process, the laser passes through the optical lens to be focused. In order to obtain a smaller feature size, the diameter of the laser spot should be as small as possible. However, smaller spot sizes correspond to smaller focal depths, which increases the difficulty of focusing. The spot size can be expressed as

(1) $$d=\frac{4M^{2}\lambda f}{\pi D}$$

where λ is wavelength, *f* is lens focal length, *D* is input beam diameter at the lens, and $M^{2}$ is the beam mode parameter.

In the desktop-level cost-effective μSL system, the focal length is about 30 mm, the depth of focus is about several tens of microns, and the diameter of the focal spot is about several microns. In order to capture such a tiny spot, it is necessary to configure a microscope head for the camera.

**Figure 2:**
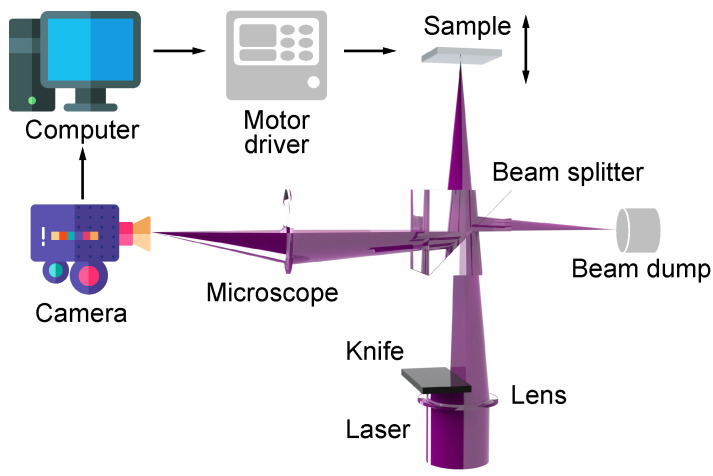
Structure design of the image-based auto-focus system.

As shown in Figure 2, the optical part of the proposed auto-focus system is composed of a visible laser source with a wavelength of 405 nm, a focus lens, a knife, a beam splitter, a microscope, a camera and a beam dump. The captured images are displayed and processed by a computer, and the motion instructions are sent to the motor drive, then the motor drives the sample to move. The entire process is fed back through the image, resulting in the sample surface being within the focal depth. To determine the relative position of the focus, the Foucault knife-edge method is used [37,38,39]. The main idea of this method is to use a “knife” to block half of the laser spot, and when the sample is located above or below the focus, the laser spot on the surface of the sample shows the left or right half circle, respectively.

The mechanism for determining the focus state in an autofocus system relies on the analysis of the laser spot’s size and distribution on the sample surface, as captured in the images. When the laser is correctly focused, the spot appears as a distinct point with a small radius and high intensity, indicating optimal concentration at the focal point. In contrast, an unfocused laser results in a larger, less intense spot. By measuring the size of this spot, one can evaluate the focus accuracy. Additionally, using a knife to partially obstruct the laser spot enables the assessment of the sample’s position relative to the focal point. The presence of the laser spot on either side indicates that the sample surface is situated on the respective side of the laser focus. This information guides the movement of the Z-motor, ensuring precise focus on the sample surface.

In the visual observation system, there are mainly bevel-axial and coaxial methods. In order to avoid distortion of laser spot images, a coaxial method is used in the proposed auto-focus system. Therefore, a transflective beam splitter is required for fabrication and observation. Not only can the laser pass through the beam splitter to fabricate the sample, but the laser spot on the sample surface can also be reflected back to the microscope and camera. A beam dump is positioned to the right of the beam splitter to eliminate unintended reflections. It should be noted that when the laser is focused on the surface of the sample, the spot image is reflected by the beam splitter and then refocused on the camera imaging surface by the microscope. To achieve this function, the position of the camera needs to be calibrated, so that the image captured by the camera can truly reflect the laser spot on the sample surface.

### 2.3. Mechanical System

**Figure 3:**
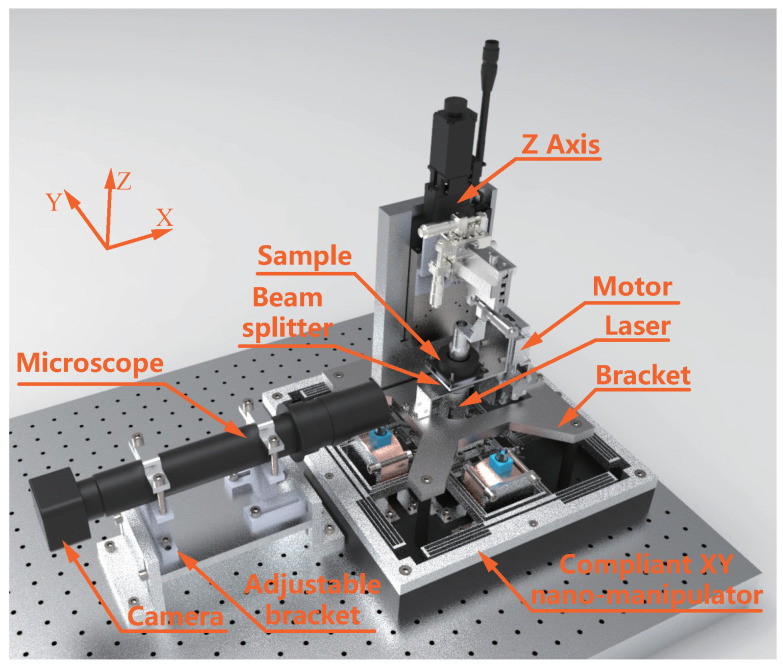
Structure of the auto-focus system and the μSL system.

The auto-focus system and the micro-stereolithography (μSL) system are illustrated in Figure 3. In this setup, the motor plays a critical role by driving the sample to move vertically along the Z axis. This movement is essential for adjusting the relative position between the sample and the laser focus, which is crucial for achieving the desired precision in the fabrication process. Proper alignment ensures that the laser beam is accurately focused on the intended area of the sample, enabling the system to create intricate microstructures with high fidelity.

To maintain the laser spot within the field of view, it is necessary to fine-tune the position of both the camera and the microscope. This fine-tuning is crucial because even minor misalignments can lead to deviations in the laser path, resulting in errors in the microstructure being fabricated. The proposed system incorporates an adjustable bracket designed to provide enhanced flexibility and precision during the alignment process. This bracket features a long-slot structure and offers 5 degrees of freedom. Specifically, these degrees of freedom include linear movement along both the X and Z axes, as well as rotational movement around the X, Y, and Z axes, respectively.

The ability to adjust the system in these multiple dimensions is particularly beneficial for accommodating variations in sample size, shape, and positioning, as well as for compensating for any potential mechanical imperfections or misalignments in the system components. By enabling precise control over the alignment and focus of the laser, this system enhances the overall accuracy and reliability of the μSL process, thereby contributing to the production of high-quality microstructures with intricate detail.

### 2.4. Control System

**Figure 4:**
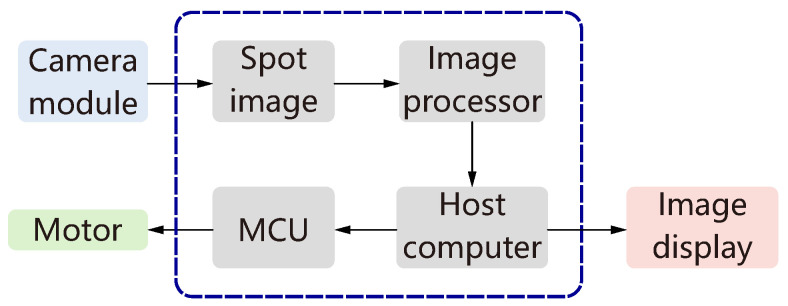
System control structure.

The control structure of the auto-focus system, as illustrated in Figure 4, integrates multiple components to achieve precise focusing of the laser spot. At the core of this system is the camera module, which plays a crucial role in capturing the laser spot image. This image is then transmitted to the host computer, where it undergoes extensive processing. The processing is guided primarily by the advanced image processing algorithm that we have developed for this specific application.

The determination of the focus state hinges on the output from this image processing algorithm. Once the focus situation has been assessed, the host computer initiates communication with the microcontroller unit (MCU). The MCU, acting as an intermediary between the host computer and the mechanical components of the system, executes commands by controlling the motor responsible for moving the camera module or lens assembly along the Z-axis.

This iterative process is repeated continuously: after each adjustment made by the motor, a new image of the laser spot is captured and processed. The cycle of image capture, processing, and motor adjustment is performed repeatedly until the optimal laser focus position is accurately determined. This systematic approach ensures that the auto-focus system achieves high precision and reliability in finding the correct focus point.

## 3. Algorithm for the Auto-Focus System

In the realm of image processing, various algorithms have been developed to facilitate the auto-focus process for general images. Notable examples include the optimized mountain-climbing method [40], quasi-conditioning [41], Prewitt operator method [41], and the sharpness-statistics-based method [42]. These methods have proven effective in traditional contexts, but the unique characteristics of the laser-focusing process demand a more specialized approach. Specifically, the auto-focus algorithm must be designed with a focus on spot size, given the distinct features of laser spot images.

Before delving into image processing, a preprocessing stage is typically employed to enhance processing efficiency and mitigate the impact of noise. The laser spot images captured by the camera inherently contain RGB (red, green, and blue) channel information. However, for our purposes, we are primarily concerned with the intensity of the laser spot. Therefore, the RGB image is converted into a grayscale image, effectively reducing the complexity of the data while retaining the crucial information. The transformation equation is as follows:

(2) $$Y=\left[\begin{array}{ccc} R & G & B \end{array}\right]\left[\begin{array}{c} 0.299\\ 0.587\\ 0.114 \end{array}\right]$$

where *Y* represents the gray value, and *R*, *G*, and *B* represent the red, green and blue channel information of the pixels in the image, respectively. This transformation ensures that the image is represented in a single channel, reflecting the overall intensity of the laser spot.

Following the grayscale conversion, binarization processing is performed to distinguish between the spot areas and non-spot areas. This step is crucial for isolating the laser spot from the background. An optimal threshold $Y_{t}$ is selected for binarization. If the grayscale value is less than the threshold, the binarization value $Y_{b}$ is set to 0, effectively classifying it as part of the background. Conversely, if the grayscale value exceeds the threshold, the binarization value $Y_{b}$ is set to 255, indicating that the pixel belongs to the laser spot, as shown in Equation (3).

(3) $$Y_{b}=\left\{\begin{array}{ccc} 0, & \; & Y<Y_{t}\\ 255, & \; & Y\geq Y_{t} \end{array}\right.$$

Determining the optimal threshold value $Y_{t}$ is crucial for effectively distinguishing objects from the background. Otsu’s method [43] is a widely employed technique for establishing the optimal threshold in the binarization process. It operates on the premise that the image comprises two pixel classes (foreground and background) and computes the threshold that minimizes intra-class variance or maximizes inter-class variance. The process consists of three steps: (1) Compute the histogram of the image; (2) Calculate the class probabilities and class means for all possible threshold values; (3) Select the threshold that minimizes the weighted sum of intra-class variances or maximizes the between-class variance.

**Figure 5:**
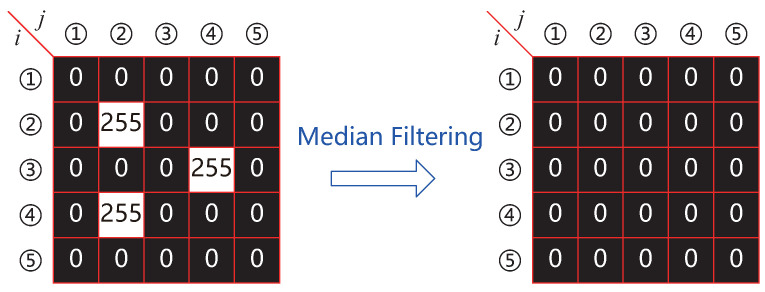
A 5×5 median filter matrix.

Camera images often suffer from noise, which can significantly hinder the accuracy of laser spot area evaluation. To address this, the binarized image undergoes filtering to remove noise while preserving critical features of the spot. Median filtering, a nonlinear technique, is widely used for this purpose. It is particularly effective in eliminating noise while maintaining edge integrity, a crucial aspect when dealing with laser spot images. In our application, a median filter utilizing a 5×5 pixel matrix is employed. The principle of this filter is illustrated in Figure 5, where the numbers in the figure denote the rows and columns of the corresponding pixels.

In noisy regions of the image, the binarization process often leaves only a few white pixels. These pixels are sorted within the 25 pixel matrix, and the median value is determined. Given that noise typically manifests as isolated white pixels, the median value is often 0, effectively setting all internal pixels to 0. This approach mitigates the impact of noise on subsequent calculations related to the laser spot area.

Once image preprocessing is completed, the next step involves extracting the contour of the laser spot. The contour is then enclosed within a minimum circle, with the radius of this circle representing the size of the laser spot. The objective is to find the center and radius of the minimum enclosing circle that contains all contour points either inside or on its boundary. To achieve this, Welzl’s recursive algorithm [44] is employed, as shown in Algorithm 1. This algorithm operates using two point sets, *P* and *R*. Here, *P* represents the set of contour points of the laser spot, while *R* is initially an empty set intended to represent the boundary of the minimum enclosing circle. The algorithm’s operation can be outlined as follows:

- Base Case: If *P* is an empty set, the algorithm terminates, and the minimum enclosing circle is defined by the points in *R*. If *R* contains exactly three points, the circle is uniquely determined by these points, enclosing all other points.
- Recursive Case: If *P* is not empty, a random point *p* is selected from *P*. A circle *D* is computed based on the current sets *P* and *R*. If *p* lies inside *D*, the algorithm proceeds without modifying *R*. If *p* lies on the boundary of *D*, *p* is added to *R*, and a new circle is computed. This process continues recursively until the minimum enclosing circle is found.

The pseudocode for this algorithm is provided below.
**Algorithm 1** Minimum enclosing circle.**Input:** Finite sets *P* and *R* of points in the plane |R|≤3. **Output: **Minimal circle enclosing *P* with *R* on the boundary. 1: **if**
*P* is empty or |R|=3 **then** 2:     **return** trivial(R) 3: **end if** 4: choose *p* in *P* (randomly and uniformly) 5: *D*← Welzl$\left(P-\left\{p\right\},R\right)$ 6: **if** *p* is in *D* **then** 7:     **return** *D* 8: **end if** 9: **return** Welzl$(P-\left\{p\right\}$, $R\cup \left\{p\right\})$

Given that the image captured by the camera often appears as a semicircle due to the Foucault knife-edge method, it is crucial to determine the direction of sample movement. An additional algorithm is designed to ascertain whether the laser spot’s semicircle lies on the left or right side of the image.

Based on the size and distribution of the laser spot, a control algorithm is developed to manage sample movement, as shown in Algorithm 2. The algorithm’s inputs include the radius *r* of the minimum enclosing circle, a given minimum radius $r_{0}$, the number of pixels in the left and right halves of the spot $n_{\mathrm{left}}$ and $n_{\mathrm{right}}$, a threshold pixel number $n_{0}$, and a small tolerance $\varepsilon_{r}$. The output is a command to the MCU, dictating the direction of movement, as shown in Algorithm 3.
**Algorithm 2** Number of pixels of left and right half spot.**Input:** The spot center (x,y) and radius *r* of the minimum enclosing circle. **Output:** Number of pixels of left and right half spot $n_{\mathrm{left}}$, $n_{\mathrm{right}}$. 1: **for all** 
i=x−r;i<x;i++
**do** 2:     **for all** j=y−r;j<y+r;j++ **do** 3:         **if** $Y_{b}=255$ **then** 4:            $n_{\mathrm{left}}++$ 5:         **end if** 6:     **end for** 7: **end for** 8: **for all** 
i=x−r;i<x+r;i++ **do** 9:     **for all** j=y−r;j<y+r;j++ **do** 10:         **if** $Y_{b}=255$ **then** 11:            $n_{\mathrm{right}}++$ 12:         **end if** 13:     **end for** 14: **end for** 15: **return** $n_{\mathrm{left}}$, $n_{\mathrm{right}}$

**Figure 6:**
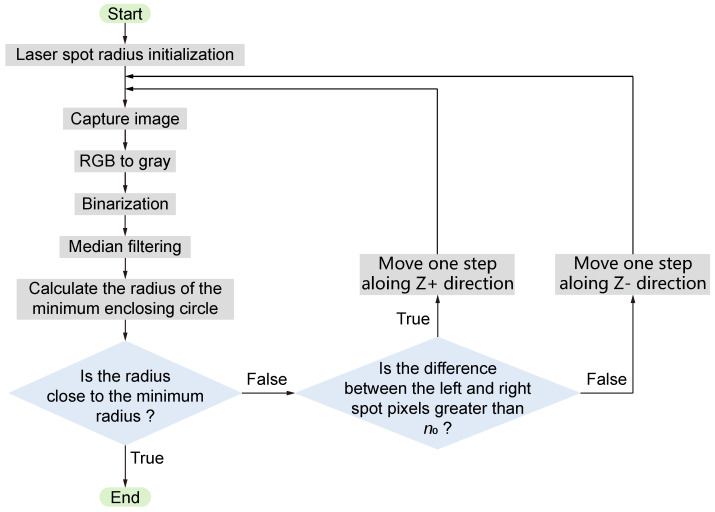
Flow chart of the algorithm.

The flowchart in Figure 6 illustrates the entire auto-focus algorithm. The process begins with initialization and preprocessing, followed by the calculation of the laser spot’s radius using the minimum enclosing circle method. Subsequently, the number of pixels in the left and right halves of the spot is determined. Based on the distribution of the laser spot, decisions are made regarding the movement of the motor, ultimately enabling the auto-focus process.

The auto-focus system described above operates by identifying the optimal focus position based on the disparity in pixel count between the left and right halves of the laser spot. While effective in many scenarios, this method primarily relies on basic image processing techniques and simple mathematical comparisons to guide the focus mechanism. However, as with any system grounded in conventional algorithms, inherent limitations arise, particularly when addressing more complex or subtle variations in the laser spot images. These limitations can result in inaccuracies, especially when the spot’s symmetry is disrupted by noise, irregularities, or environmental factors.
**Algorithm 3** Command to the MCU.**Input:** The radius *r* of the minimum enclosing circle, a given minimum radius $r_{0}$, the number of pixels of left and right half spot $n_{\mathrm{left}}$, $n_{\mathrm{right}}$, a given number $n_{0}$, and a given small number $\varepsilon_{r}$. **Output:** Command to the MCU: upward, downward and stop. 1: **if** $r-r_{0}>\varepsilon_{r}$ and $n_{\mathrm{left}}-n_{\mathrm{right}}>n_{0}$ **then** 2:     **return** upward 3: **end if** 4: **if** $r-r_{0}>\varepsilon_{r}$ and $n_{\mathrm{left}}-n_{\mathrm{right}}\leq n_{0}$ **then** 5:     **return** downward 6: **end if** 7: **if** 
$r-r_{0}\leq \varepsilon_{r}$ 
**then** 8:     **return** stop **end if**

**Figure 7:**
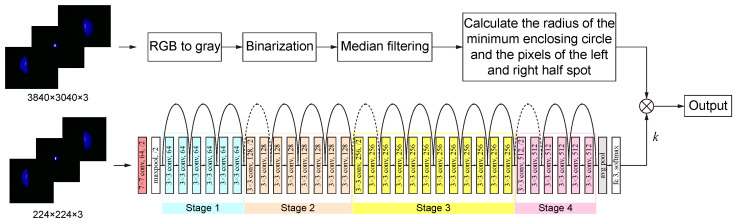
Image processing algorithm based on deep learning.

Since the pixel difference between the left and right halves of the laser spot near the focus is often minimal, relying solely on pixel distribution to determine the focus position may lead to deviations. This issue can be resolved by applying the proposed image processing algorithm, which utilizes deep learning techniques to process the differences between the left and right spots. To address these challenges and enhance the system’s performance, we propose the integration of a classification algorithm based on deep learning, as illustrated in Figure 7.

The proposed algorithm employs deep learning to autonomously classify the focus position of the current sample. The deep learning approach is grounded in the ResNet 34 classification model [45], which classifies the laser spots on the sample surface based on their characteristics, thus automatically determining the laser focus position. It comprises four stages and a total of 34 layers. The network architecture is structured as a series of residual blocks, each containing shortcut connections that bypass one or more layers. These shortcut connections enable the network to “skip” certain layers, facilitating the learning of identity mappings and enhancing gradient propagation. Unlike traditional methods, which depend on predefined rules and thresholds, deep learning models can learn from extensive datasets, identifying intricate patterns that may not be immediately apparent. The internal structure of the deep learning network consists of a series of convolutional layers arranged into residual blocks. Each block contains two or more convolutional layers, followed by batch normalization and ReLU activation. The network begins with a single convolutional layer and a max-pooling layer, then progresses through four main stages, where the number of residual blocks and filters increases. These stages transform the input image through increasingly complex feature extraction processes, culminating in a global average pooling layer and a fully connected layer for classification. By training the algorithm on a diverse set of laser spot images, the system gains a sophisticated understanding of how different focus positions correspond to specific features in the images.

To determine the laser’s position relative to the focus, the pixel difference is multiplied by a significant factor *k*. A set threshold can then be used to ascertain the laser focus point and the sample surface by evaluating the pixel differences between the left and right sides of the laser spot. Deep learning, with its capability to model complex patterns and relationships within data, provides a robust framework for enhancing the precision and adaptability of the auto-focus system.

The deep learning method, in particular, can be designed to analyze a variety of laser spot characteristics, including shape, intensity distribution, and symmetry. The light spots collected by the same camera are consistent and repeatable, which allows the algorithm to learn effectively from the data. Through this analysis, the algorithm can make more informed decisions regarding the direction in which the motor should move to achieve optimal focus. Additionally, the deep learning algorithm exhibits a strong generalization ability, ensuring the correctness of the classification results, even when faced with irregular laser spots or distortions in the imaging environment that may complicate the focusing process.

The integration of deep learning into the auto-focus system represents a significant advancement in both accuracy and reliability in focus determination. By providing a more direct and precise evaluation of motor movement, this approach not only refines the auto-focus process but also enhances the overall system performance in challenging and dynamic environments. This advancement is particularly critical in applications requiring high precision, such as high-resolution imaging, laser processing, and other fields where even minor focus deviations can result in significant errors or quality issues. The incorporation of a deep learning-based classification algorithm into the auto-focus system marks a substantial improvement over traditional methods. By leveraging the power of deep learning, the system achieves greater accuracy and adaptability, ensuring optimal focus under diverse conditions and contributing to more reliable and consistent outcomes in practical applications.

## 4. Prototype and Experimental Results

To validate the structure and algorithm of the proposed image-based auto-focus system, a comprehensive prototype system is developed and subjected to rigorous experimental testing. The results of these tests are discussed in detail to demonstrate the effectiveness and precision of the system.

### 4.1. Prototype

The μSL system is primarily composed of several critical components: a nano-manipulator for planar motion, a laser source for precise fabrication, a sample for testing, and a bracket to ensure stability during operations. The auto-focus system is comprised of a beam splitter, a camera for capturing the laser spot, an adjustable bracket, a motor for movement, a motor driver, a host computer for control and data processing, and an MCU for real-time system management.

The beam splitter plays a crucial role by directing the laser spot on the sample surface to the camera, enabling real-time image capture. The host computer displays the focus status continuously, conducts image processing, and communicates the processing results to the MCU which, in turn, controls the motor to adjust the focus automatically. This integrated system ensures that the focus is maintained accurately and consistently throughout the operation.

### 4.2. Calibration

Calibration is an essential step in ensuring the accuracy of the auto-focus system. The imaging process involves focusing on the original images, which necessitates precise alignment of the camera and microscope to ensure that the laser spot displayed in the image correctly represents the sample surface.

**Figure 8:**
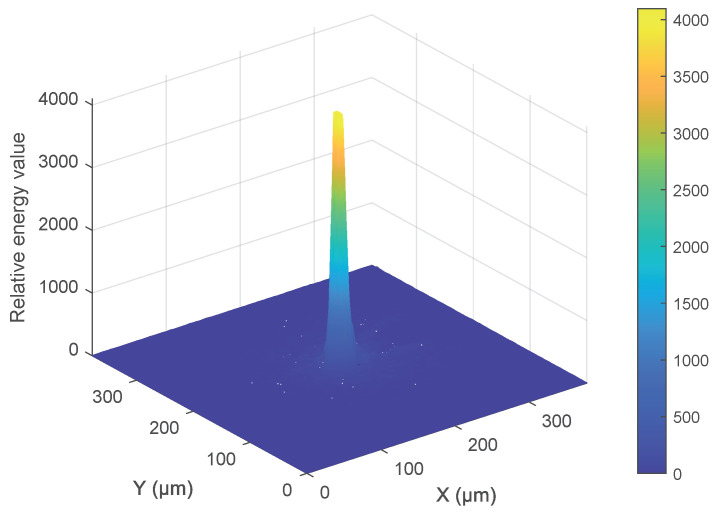
Energy distribution of the laser spot.

During the calibration process, a laser beam analyzer is positioned at the sample location. Figure 8 shows the energy distribution of the laser spot. The Z-motor is then adjusted to achieve the highest concentration of spot energy as displayed by the laser beam analyzer. Following this, the positions of the camera and microscope are fine-tuned. Calibration is considered complete when the laser spot captured by the camera is at its smallest, indicating that the camera is correctly aligned with the sample surface. This calibration ensures that the system can accurately detect and focus on the laser spot, which is critical for the precise operation of the auto-focus mechanism.

### 4.3. Experimental Results

**Figure 9:**
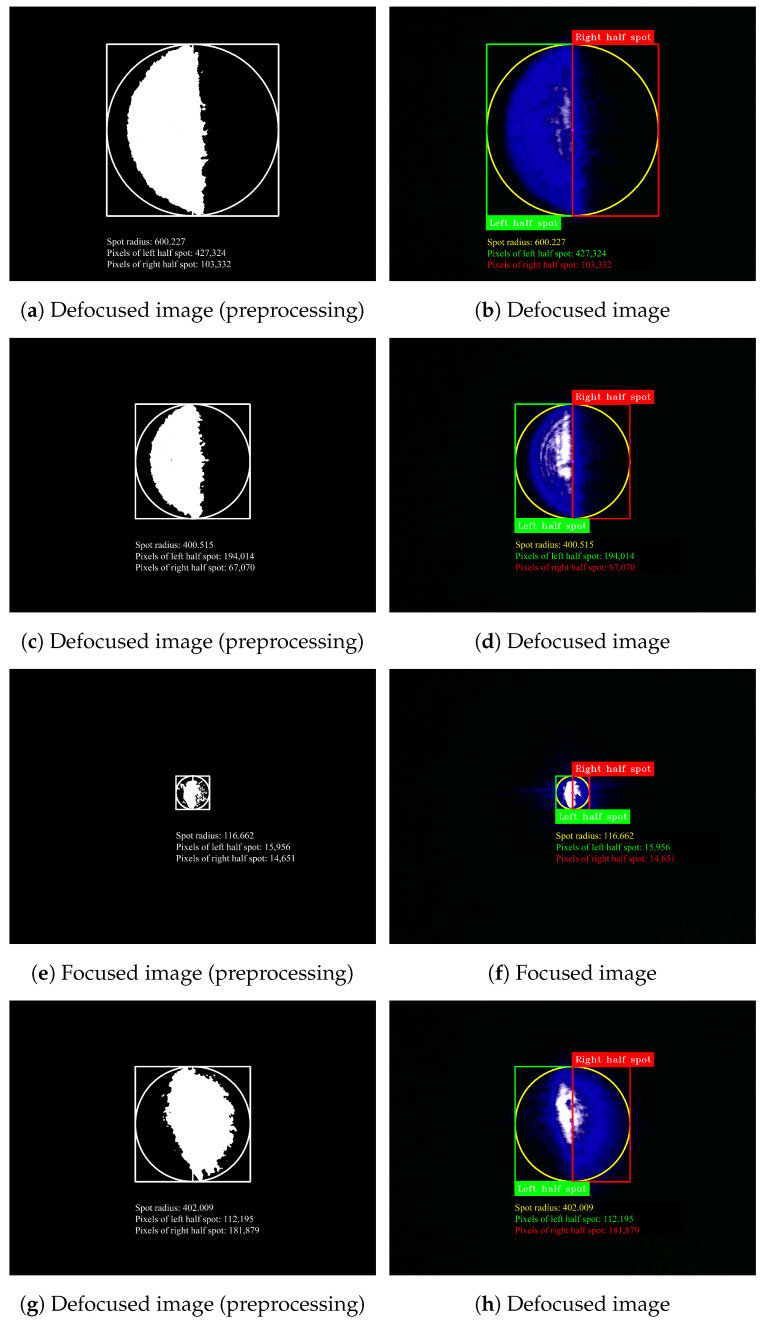

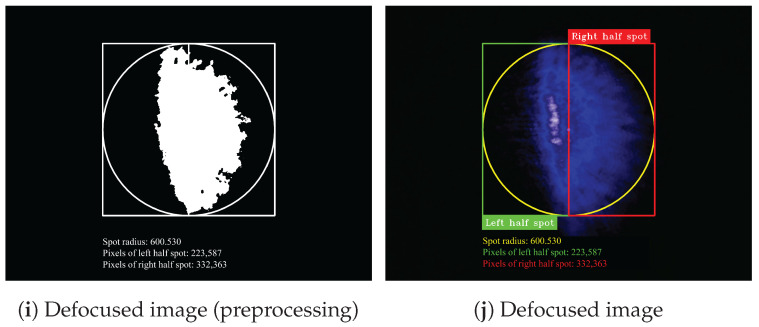
Laser spot images at different positions.

After successful calibration, a series of experiments were conducted to evaluate the performance of the proposed auto-focus system. The results of these experiments, as depicted in Figure 9, provide a detailed analysis of the system’s effectiveness under various conditions.

Utilizing the Foucault knife-edge method, the laser spot is visualized as left and right halves, which appear at the bottom and top of the focus, respectively. The proposed algorithm determines the relative position of the current location and focus by analyzing the radius size and pixel distribution of the laser spot. By adjusting the relative angle between the knife and the camera, we ensure that the dividing line between the two halves remains vertical. This approach, in addition to software rotation, effectively enhances the processing speed of our algorithm. Notably, as shown in Figure 9, the proposed method allows for a certain tilt, thereby simplifying system layout and operation, while demonstrating that even if there is some offset in the knife or camera, the result of laser focusing remains unaffected.

**Figure 10:**
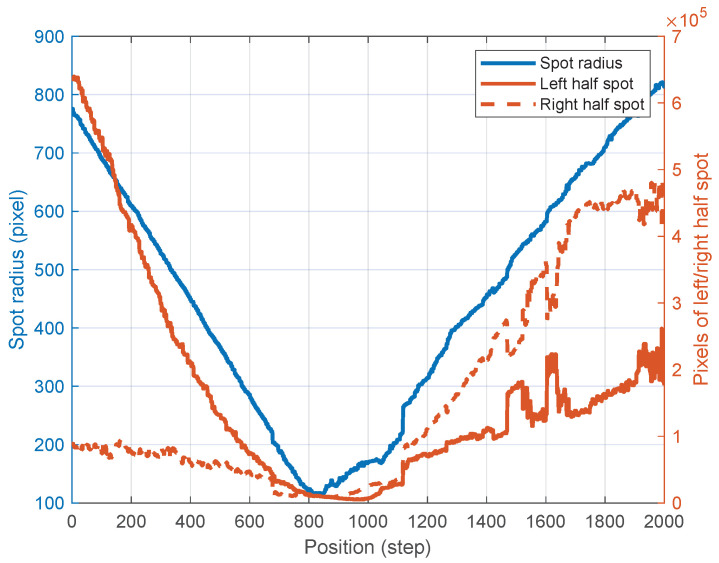
Spot radius and pixels of left/right half spot.

During these experiments, the spot radius and the pixel counts of the left and right half-spots at various positions were meticulously recorded (Figure 10). The data show a significant decrease in spot radius during the focusing process, accompanied by a convergence in the pixel counts of the left and right spots. This convergence is indicative of the system’s ability to achieve precise focus.

**Figure 11:**
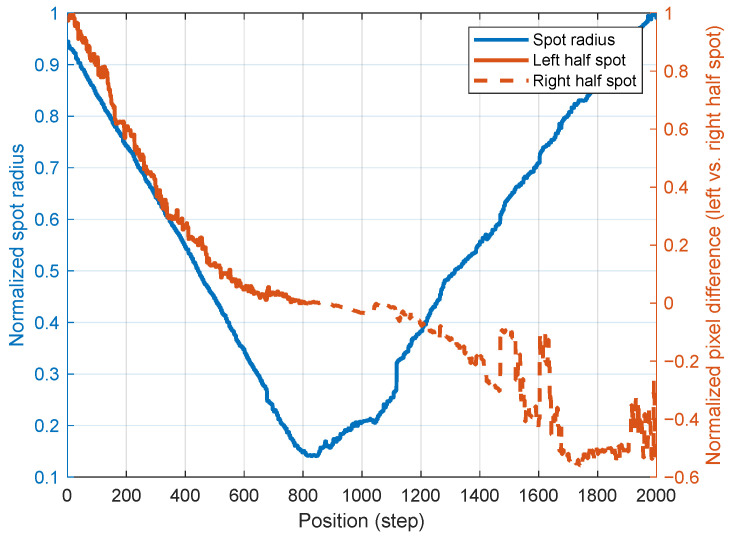
Relative laser spot radius and difference between pixels of left and right half spot.

To further validate the effectiveness of the proposed algorithm, normalization of the data was performed. This process involved dividing the laser spot radius and the pixel difference between the left and right half-spots by their maximum values (Figure 11). The consistency of the laser spot radius within the minimum radius field serves as a criterion for determining the completion of the auto-focus process. Additionally, the distribution of laser spot pixels is used as a basis for adjusting the sample’s position, ensuring optimal focus throughout the process.

**Figure 12:**
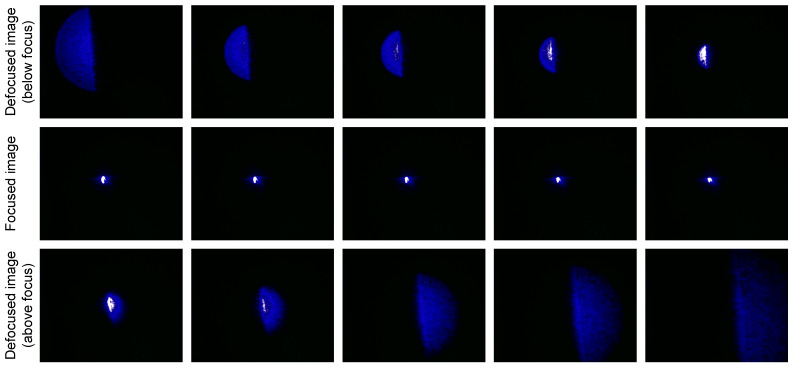
Images in the train set.

In order to validate the proposed deep learning-based image processing algorithm, we assembled a dataset of 4690 images capturing the laser focusing process. This dataset was divided into three subsets: the training set, validation set, and test set, in proportions of 60%, 20%, and 20%, respectively. Figure 12 presents representative images of lasers in various states from the training set.

**Figure 13:**
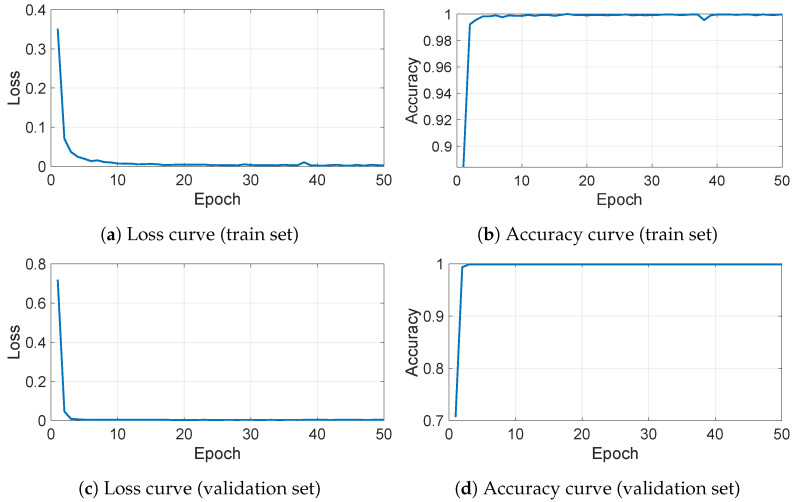
Model loss and accuracy curves for 50 epochs.

All training and validation processes were carried out using TensorFlow (GPU version 2.4.0). The model was trained for 50 epochs, with a batch size of 8. Training and deployment were performed on a 64-bit workstation with 32 GB of RAM, AMD Ryzen^TM^ 9 5900X CPU (12-core, 3.70 GHz), and an NVIDIA^®^ GeForce^®^ GTX^TM^ 3080 GPU. Figure 13 illustrates the loss and accuracy curves for both the train and validation sets during model training. The model achieved convergence in fewer than 10 epochs and consists of 21,303,235 parameters, occupying 81.2 MB.

**Figure 14:**
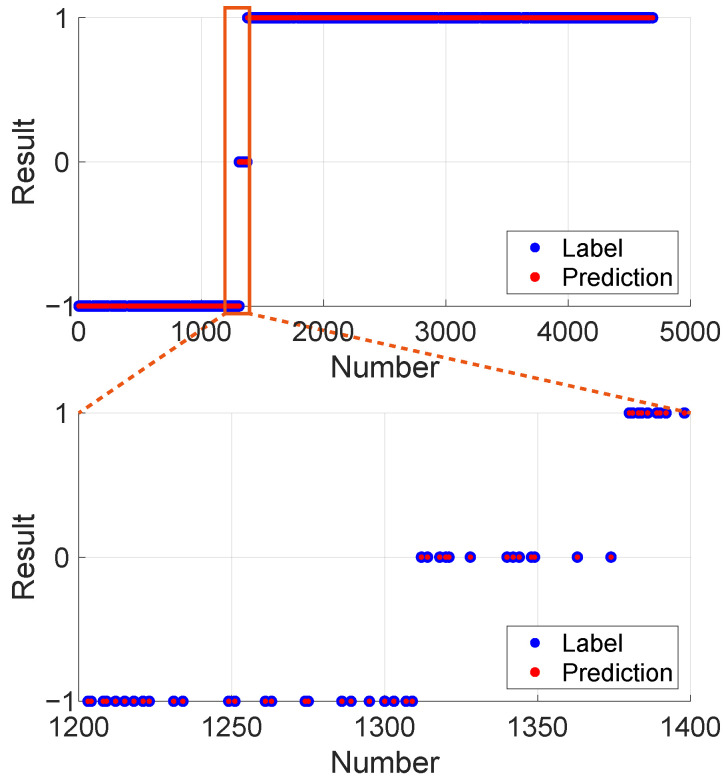
Prediction results for images in the test set.

To further evaluate the effectiveness of the proposed algorithm and the trained model, the laser spot images from the test set are subjected to evaluation. The results, presented in Figure 14, show that the spot below focus is labeled as −1, the spot at focus as 0, and the spot above focus as 1. Blue points represent the true labels, while red points indicate the predicted results. To enhance clarity, the section between 1200 and 1400 on the horizontal axis is magnified for detailed visualization. The classification performance on the test set demonstrates that all images are correctly classified.

**Figure 15:**
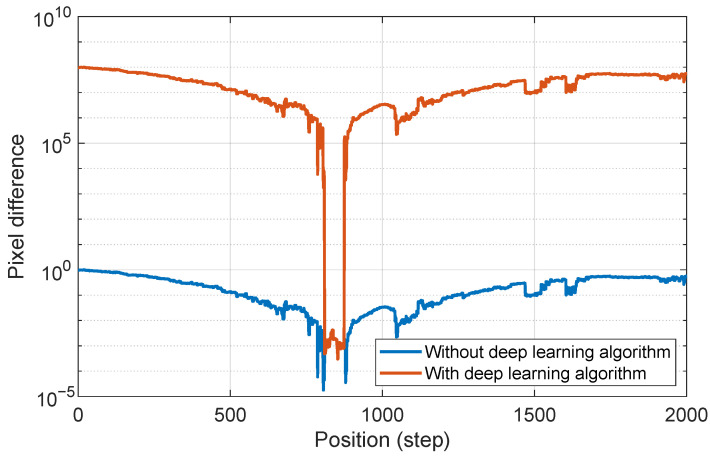
Comparison of the pixel differences with and without the proposed deep learning algorithm.

Due to the minimal difference in pixel count between the left and right laser spots near the focal point, determining the focal position based solely on pixel distribution can lead to inaccuracies. This issue can be mitigated by applying the proposed deep learning-based image processing algorithm, which analyzes the differences between the left and right spots. For distinguishing the laser’s position above (1) and below (−1) the focal point, the pixel difference is amplified by a factor (e.g., $10^{8}$), as depicted in Figure 15. Without the deep learning algorithm, determining an optimal threshold for position classification is challenging. However, with the proposed approach, selecting an appropriate threshold to determine the relative position of the laser focus with respect to the sample surface becomes straightforward.

Regarding the accuracy of the optimal focus position identification, we conducted a series of experiments to validate our methodology. The results demonstrate a strong correlation between the measured disparities and the actual focus positions. Our approach achieved an accuracy of approximately ±1 µm under controlled conditions, which is adequate for applications requiring precise focus adjustments, especially when compared to laser focus depths of tens of microns. Furthermore, the system has been tested under various conditions, consistently yielding reliable and accurate outcomes.

**Figure 16:**
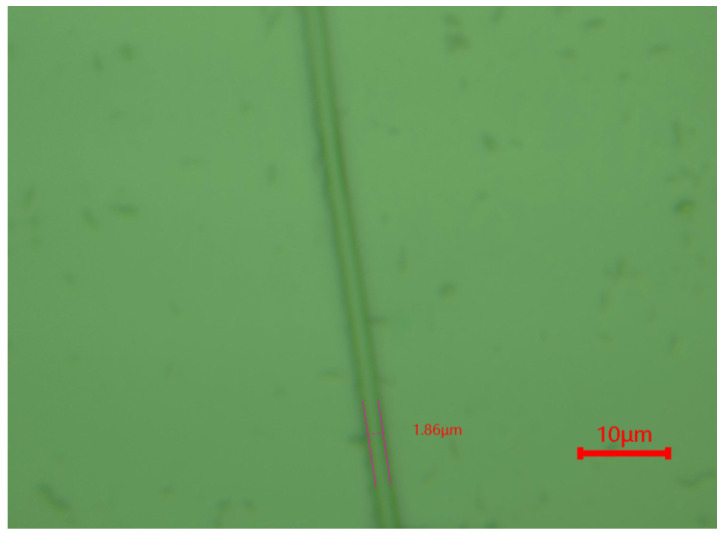
Sample of micro-stereolithography fabrication.

Finally, a sample was fabricated using the μSL system to demonstrate the practical application of the auto-focus system. As shown in Figure 16, the processed line width was approximately 1.86 µm, showcasing the high precision and reliability of the system. The successful development and integration of the image-based auto-focus system with the μSL system provide a robust technical foundation for micron-level laser direct writing processes, paving the way for future advancements in this field.

## 5. Conclusions

In this paper, we have developed an image-based auto-focus system supporting micro-stereolithography. The optical path and mechanical structure of the desktop-level cost-effective auto-focus system are built, including a beam splitter, a camera, an adjustable bracket, a motor, a motor driver, a host computer and an MCU. Furthermore, the Foucault knife-edge method is employed to determine the relative position of the current location and the focal point of the sample. Moreover, we have proposed a deep learning-based laser spot image processing algorithm based on the laser spot size and pixel distribution for autofocus. Finally, it is noteworthy that the experimental results strongly validate the effectiveness of the proposed image-based auto-focus system.

Future work will focus on integrating our image-based auto-focus system with advanced techniques for optimizing 3D imaging applications, particularly in single-particle tracking and super-resolution microscopy. Building on the framework proposed in the previous study, we plan to incorporate pupil-plane modulation to maximize the information content of our system.

## Data Availability

The original contributions presented in the study are included in the article, further inquiries can be directed to the corresponding authors.
